# Long-term cognitive outcome in adult survivors of an early childhood posterior fossa brain tumour

**DOI:** 10.1007/s10147-020-01725-7

**Published:** 2020-07-08

**Authors:** Adam P. Wagner, Cliodhna Carroll, Simon R. White, Peter Watson, Helen A. Spoudeas, Michael M. Hawkins, David A. Walker, Isabel C. H. Clare, Anthony J. Holland, Howard Ring

**Affiliations:** 1 National Institute for Health Research (NIHR) Applied Research Collaboration (ARC) East of England, Cambridge, UK; 2 Norwich Medical School, University of East Anglia, Norwich, UK; 3 Department of Psychiatry, University of Cambridge, Cambridge, UK; 4 Kent Clinical Neuropsychology Service, Kent and Medway NHS and Social Care Partnership, Kent, UK; 5 MRC Biostatistics Unit, University of Cambridge, Cambridge, UK; 6 MRC Cognition and Brain Sciences Unit, University of Cambridge, Cambridge, UK; 7 Paediatric Neuroendocrinology, Great Ormond Street and University College London Hospitals, London, UK; 8 Centre for Childhood Cancer Survivor Studies, Institute of Applied Health Research, Robert Aitken Building, University of Birmingham, Birmingham, UK; 9 Children’s Brain Tumour Research Centre, Faculty of Medicine and Health Sciences, University of Nottingham, Nottingham, UK; 10 Cambridge Lifespan Autism Spectrum Service, Cambridgeshire & Peterborough NHS Foundation Trust, Cambridge, UK; 11 Cambridgeshire and Peterborough NHS Foundation Trust, South Team, Comberton Road, Toft, Cambridgeshire, CB23 2RY UK

**Keywords:** Posterior fossa, Childhood brain tumour, Survivorship, Cognition

## Abstract

**Purpose:**

Posterior fossa brain tumours (PFT) and their treatment in young children are often associated with subsequent cognitive impairment. However, reported follow-up periods rarely exceed 10 years. This study reports very long-term cognitive consequences of surviving an early childhood PFT.

**Methods:**

62 adult survivors of a PFT, ascertained from a national register, diagnosed before 5 years of age, and a sibling control, received a single IQ assessment an average of 32 years (range 18–53) after initial diagnosis, using the Weschler Abbreviated Scale of Intelligence. Regression models were fitted to survivor–sibling pair differences on verbal and performance IQ (VIQ and PIQ) scores to investigate whether increasing time between PFT diagnosis and follow-up IQ assessment contributed to survivor–sibling IQ differences.

**Results:**

At follow-up, survivors had, on average, VIQ 15 points and PIQ 19 points lower than their siblings. There was no significant effect of time since diagnosis on survivor–sibling VIQ difference. Survivors who received radiotherapy showed no significant effect of time since diagnosis on survivor–sibling PIQ difference. Survivors who did not receive radiotherapy demonstrated a trend for it to reduce.

**Conclusions:**

VIQ and PIQ deficits persist in adulthood, suggesting the effect of a fixed injury imposing on cognitive development, rather than an ongoing pathological process.

**Implications for cancer survivors:**

The findings will help parents and others supporting survivors of an early life PFT to identify and plan for possible cognitive outcomes, and highlight the importance of early interventions to optimize cognitive function during the developmental period.

**Electronic supplementary material:**

The online version of this article (10.1007/s10147-020-01725-7) contains supplementary material, which is available to authorized users.

## Introduction

Posterior fossa brain tumours (PFT) and their treatment in children can be associated with subsequent cognitive impairment [1–4]. Rates of survival from childhood brain tumours have been increasing but tumour management tends to be more challenging in younger children and it is amongst these survivors that greater cognitive morbidity is observed [5–9]. Factors associated with increased risk of later cognitive decline include, most powerfully, radiotherapy [10–13].

Longer periods since diagnosis are associated with more impaired intellectual functioning [7, 9, 10, 14, 15]. However, whilst many studies have described short- and medium-term cognitive outcomes in survivors of a childhood PFT (see reviews by Robinson et al. [3], de Ruiter et al. [10], Nathan et al. [16]), few have investigated long-term cognitive outcomes in later adulthood of PFT survivors diagnosed in early childhood [3, 6, 9, 11].

This study reports long-term cognitive consequences of surviving an early childhood PFT. The results should be relevant for survivors, their families and their clinicians, and for services supporting adults with a history of an early childhood PFT.

## Methods

### Study design

Adult survivors of a childhood PFT, diagnosed before the age of 5 years, were identified from the UK National Registry of Childhood Tumours (UK NRCT), which holds clinical data going back as far as the 1940s. We aimed to recruit a complete as possible sample across England to participate in a single face-to-face assessment including an established IQ measure. To provide an approximate indication of how pre-morbid intellectual ability may have developed in these survivors if they had not had a brain tumour, we measured the IQ of a non-affected sibling of each survivor. This work was part of a wider project that examined mental health outcomes in adult survivors of early childhood PFT [17].

### Eligibility criteria

**Fig. 1:**
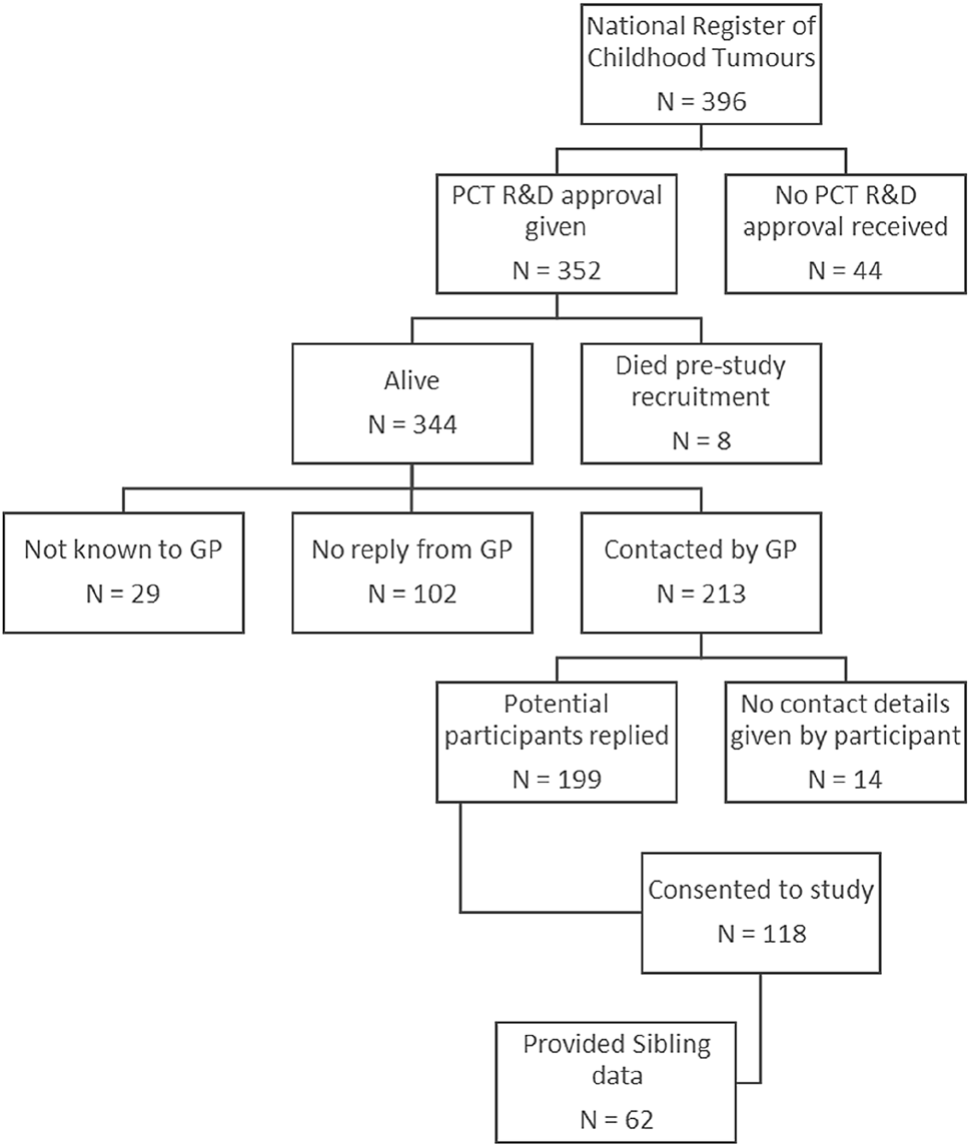
Flow chart of recruitment. Adapted from Carroll et al. [17]

Adults aged at least 18 years at follow-up who had been diagnosed with a PFT before the age of 5 years, between the years 1940 and 1991, were identified from the UK NRCT and recruited through their General Practitioners (GPs). In the main analyses reported here, the adult survivor group comprised all those for whom a sibling provided comparison IQ data (Fig. 1). Siblings were recruited through the index participants, with one sibling recruited for each index participant. Where more than one sibling was available, the one closest in age to the index participant was recruited.

### Approvals and consent

Approval was given by the Cambridgeshire 2 Research Ethics Committee and UK National Information Governance Board. Informed consent was obtained from all individual participants included in the study who had capacity to consent. Participants lacking capacity were included where agreement was gained from family or carers, as required by the *Mental Capacity Act (England and Wales) 2005*.

### Assessments

IQ was measured using the Wechsler Abbreviated Scale of Intelligence (WASI) [18]. This is a standardised, psychometrically robust, brief measure of intelligence assessing verbal knowledge, verbal reasoning, visual information processing, and visual perception, through the use of four subtests: vocabulary and similarities to measure verbal IQ (VIQ), block design and matrix reasoning to measure performance IQ (PIQ) [18]. The WASI correlates highly with the equivalent full Wechsler Adult Intelligence Scale (WAIS-III, [19]; WASI and WAIS-III correlations [18]: VIQ 0.88, PIQ 0.84), but makes fewer demands on participants.

A structured psychiatric assessment was also undertaken, results from which have been published previously [17].

### Tumour registry data

For each survivor, age at diagnosis, sex, tumour type and treatment received were extracted from the NRCT. Available treatment details varied between participants, so cranial radiotherapy, chemotherapy and surgery descriptions were dichotimised to confirmed use, or not, of the treatment. Tumour types were condensed into astrocytoma, medulloblastoma or ‘other’.

### Statistical analysis

The main aim is to investigate whether increasing time since tumour diagnosis affects VIQ and PIQ outcomes in adulthood of survivors of an early childhood PFT. We also investigate if the changes over time differ for those who had received radiotherapy.

Univariate group comparisons tested differences between groups of survivors and their siblings using Fisher’s exact test and *t* tests as appropriate. Subsequently, within the survivors, we investigated relationships between VIQ and PIQ, cancer treatment received, age at diagnosis and sex, using Fisher’s exact test and *t*-tests as appropriate. Finally, regression models further investigated differences in IQ between survivors and their siblings.

Odds ratios (ORs) and Pearson’s correlation coefficient (*r*) are used as measures of effect size. In places, Pearson’s *r* was calculated from *t*-statistics, as described in Field et al. [20], using a formula from Rosenthal and Rubin [21]. Effect sizes are interpreted according to the benchmarks in Cohen [22].

### Statistical analysis: regression modelling

The difference between survivors’ and their siblings’ VIQ and PIQ scores was analysed using generalised additive models (GAMs); these were fitted using the mgcv package (see Wood [23]) in the statistical software R.

In each model, the sibling’s IQ score is included as a covariate to act as a proxy for unmeasured confounders (elements of environment, including shared family environment and genetics). We also include a quadratic form of the IQ measures as a further covariate in case the relationship with unmeasured confounders is not linear. Both linear and quadratic forms of the sibling measures are centered (using a mean of 100).

Further covariates (levels of categorical variables denoted in parentheses) include: survivor sex; sibling sex (same, different); epilepsy (present, absent); (centred) time in years since diagnosis; age in months at diagnosis; tumour type (astrocytoma, medulloblastoma, other); radiotherapy (no confirmed treatment, confirmed radiotherapy); surgery (no confirmed surgery, confirmed surgery). GAMs allow fitting of a penalized regression spline to time since diagnosis, the covariate of primary interest, allowing more flexible relationships (rather than a straight line); Supplementary materials present an analysis restricted to linear relationships for comparison.

We consider five interactions (denoted ‘ × ’): survivor sex × sibling sex; survivor sex × radiotherapy (explores how the IQ of female survivors is affected by radiotherapy); radiotherapy × surgery; tumour type × radiotherapy; and time since diagnosis × radiotherapy (explores how the effect of radiotherapy differs over time, within GAM framework this results in separate curves for each level of radiotherapy). Within each set of GAMs, models were compared using the second-order Akaike Information Criterion (AICc [24, 25], a small sample size version of the standard AIC), where lower values indicate a better fitting model, to investigate the importance of the five interactions.

## Results

### Recruitment

A potential maximum of 396 survivors were initially identified from the UK NRCT. Of these, 213 people could be contacted via their GP and 118 took part, representing a 55% response amongst contactable survivors (Fig. 1). A non-responder analysis demonstrated that demographic and cancer characteristics were similar between these 118 participants and those not participating (see Supplementary Materials in Carroll et al. [17]). Of the 118 participant survivors, 62 with a sibling who provided comparison data are reported on here. There was no indication of selection bias comparing suvivors without a recruited sibling (see Supplementary material).

### Survivors: demographics and other characteristics

Mean age at data collection was similar across females (35.6) and males (34.2) (*t*(52) = − 0.545, *p* = 0.588; *r* = 0.075) as was years of education in excess of 10 years for females (3.1) and males (3.2) (*t*(42) = 0.190, *p* = 0.850; *r* = 0.029). Twelve survivors had epilepsy and epilepsy history was unknown for one individual.

### Survivors: diagnosis and treatment details

Details of survivors’ age at diagnosis, tumour, and treatment are in Table 1. There was little difference in the age at which males (39.6 months) and females (42.0) had been diagnosed (*t*(51) = − 0.714; *p* = 0.478; *r* = 0.100). Tumour type did not affect age of diagnosis (*F*(2,59) = 0.979, *p* = 0.382). There was no difference between sexes in the proportion that received surgery (Fisher’s exact test: *p* = 1.000; OR = 1.127). For further analysis of variables associated with surgery, see Supplementary Materials.

**Table 1:** Survivor diagnosis and treatment information

Diagnosis/treatment attribute		Statistics/*N*
Age at diagnosis	Months	Mean = 40.5, median = 44.0, SD = 13.3, range = 4–59
Time since diagnosis	Years	Mean = 31.3, median = 29.5, SD = 9.9, range = 18–53
Tumour type	Astrocytoma	40
	Medulloblastoma	15
	Other	7
Radiotherapy Chemotherapy	No confirmed treatment	26
	Confirmed radiotherapy	31
	Confirmed radiotherapy and chemotherapy	5
Surgery	No recorded surgery	11
	Biopsy only	1
	Palliative surgery	2
	Removal (unspecified type) of primary tumour	8
	Partial removal of primary tumour	18
	Total removal of primary tumour	22

*SD* standard deviation

Astrocytoma was the most common tumour type (*n* = 40) and included: fibrillary—3; pilocytic—28; NOS—9. All the medulloblastoma (*n* = 15) were recorded as ‘medulloblastoma, NOS’. The ‘Other’ group of tumours included: tumour cells—1; subependymoma—1; ‘ependymoma, NOS’—4; ‘spongioblastoma, NOS’—1.

Age at diagnosis was not associated with whether radiotherapy was received (*t*(47) = − 0.314; *p* = 0.755; *r* = 0.045). While not statistically significant (*t*(49) = − 1.513; *p* = 0.137), those who received radiotherapy had been diagnosed further into the past than those who had not (mean time since diagnosis (years): received radiotherapy = 32.9, no radiotherapy = 29.0), with a small to medium effect (*r* = 0.212). There was little difference between sexes in the proportion who received radiotherapy (Fisher’s exact test: *p* = 0.429; OR = 0.589). Radiotherapy was more common for medulloblastomas (radiotherapy: medulloblastoma = 87%, astrocytomas = 48%, other = 57%; Fisher’s exact test: *p* = 0.025). Five survivors received chemotherapy in addition to radiotherapy. No participant had chemotherapy without radiotherapy.

There was no significant difference in the proportion having surgery between those treated without radiotherapy (88%) and with radiotherapy (78%) (Fisher’s exact test: *p* = 0.332; small to medium effect,OR = 2.16). Around 45% (28/62) of survivors had radiotherapy and surgery.

### Demographic comparisons between survivors and siblings

Descriptions of the 62 tumour survivors and their sibling comparators are given in Table 2.

**Table 2:** Differences between the 62 survivor and sibling pairings

Attribute	Survivors			Siblings			Survivor v. sibling (unadjusted) comparisons
	Mean	SD	Range	Mean	SD	Range	
Sex: females	*n* = 24	–	–	*n* = 36	–	–	Fisher's exact test, *p* = 0.048, OR = 0.459
Employed	*n* = 39^a^	–	–	*n* = 48^b^	–	–	Fisher's exact test, *p* = 0.196, OR = 1.890
Age (months) at data collection	34.8	10.1	19–57	35.0	10.8	19–59	*t*(61) = − 0.502, Dif = − 0.3 (95% CI − 1.4, 0.8), *p* = 0.617, *r* = 0.064
Age (years; deciles)							
19–29	*n* = 26	–	–	*n* = 23	–	–	Fisher's exact test, *p* = 0.714
30–39	*n* = 16	–	–	*n* = 19	–	–	
40–49	*n* = 15	–	–	*n* = 12	–	–	
50–59	*n* = 5	–	–	*n* = 8	–	–	
Years beyond compulsory education	3.2^c^	2.2	0–7	4.3^b^	2.5	0–7	*t*(47) = − 3.013, Dif = − 1.3 (95% CI − 2.1, − 0.4), *p* = 0.004, *r* = 0.402
Verbal IQ	88.3	18.1	55–121	103.7	12.0	77–127	*t*(61) = − 6.774, Dif = − 15.4 (95% CI − 19.9, − 10.8), *p* < 0.001, *r* = 0.655
Performance IQ	91.1^d^	20.3	55–129	110.0	12.3	83–134	*t*(60) = − 6.982, Dif = − 19.1 (95% CI − 24.6, − 13.7), *p* < 0.001, *r* = 0.670

Paired *t* tests are used for comparing continuous variables between groups. One survivor could not complete PIQ subtests due to vision problems, and so has no corresponding PIQ score

^a^*N* = 56

^b^*N* = 59

^c^*N* = 50

^d^*N* = 61

### IQ comparisons between survivors and siblings

Table 2 provides the VIQ and PIQ summary scores. The survivors’ VIQ scores were significantly lower on average than their siblings, by a mean difference of 15 points (*p* < 0.001; *r* = 0.655), and their PIQ scores by a mean difference of 19 points (*p* < 0.001; *r* = 0.670). However, these mean group differences, as displayed in Supplementary Figure S1, include a wide range of differences in IQ scores between survivors and siblings.

Supplementary Table 1 provides the IQ sub-test scores for survivors and siblings, demonstrating similar magnitudes of difference between sibling and survivor across all subtests. Unadjusted group IQ scores separated by sex are detailed in Supplementary Table S2.

Mean VIQ and PIQ for different tumour types, along with sibling comparison IQ scores, are reported in Supplementary Table 3. For survivors, means of VIQ and PIQ by tumour type are similar. When compared with their siblings, those with medulloblastomas score worse on VIQ (mean difference compared to siblings: medulloblastomas = − 20.7, astrocytomas = − 13.5 and ‘other’ tumour type = − 14.7); however, this variation in difference is small (*r* ≈ 0.7 versus *r* ≈ 0.6).

### GAM: verbal IQ

The VIQ GAM with lowest AICc is reported in Table 3 and includes two interactions (survivor sex × sibling sex; survivor sex × radiotherapy). In this model *n* = 61 (one participant has missing information about epilepsy), adjusted *R*^2^ = 0.32 and diagnostic plots suggest no problems with model fit.

**Table 3:** The fit of the generalised additive models (GAMs) relating verbal IQ (VIQ) and performance IQ (PIQ) difference (survivor IQ-sibling IQ) to the covariates

Variable	Categorical level	VIQ (*n* = 61; adjusted *R*^2^ = 0.32)					PIQ (*n* = 60; adjusted *R*^2^ = 0.41)				
		*b*	95% CI		*p* value	*r*	*b*	95% CI		*p* value	*r*
Intercept	–	− 18.9	− 40.2	2.5	0.090	0.247	0.0	− 23.8	23.7	0.997	0.000
Age at diagnosis (months)	–	− 0.2	− 0.5	0.2	0.379	0.130	0.3	0.0	0.7	0.074	0.258
Tumour type^a^	Astrocytoma	4.2	− 6.3	14.8	0.437	0.115	− 3.0	− 14.9	8.8	0.620	0.073
	Other	9.8	− 4.9	24.5	0.199	0.188	− 5.0	− 22.1	12.2	0.572	0.083
Radiotherapy^b^	Confirmed radiotherapy	2.7	− 8.5	13.8	0.641	0.069	− 18.0	− 27.7	− 8.3	**<0.001**	0.468
Epilepsy^c^	Present	− 7.4	− 17.6	2.8	0.160	0.205	− 9.5	− 21.2	2.2	0.117	0.227
Surgery^d^	Confirmed surgery	15.4	3.8	27.0	***0.013***	0.357	4.3	− 7.8	16.3	0.491	0.101
Survivor sex^e^	Female	− 2.6	− 16.1	11.0	0.711	0.055	− 16.8	− 26.0	− 7.5	**<0.001**	0.461
Sibling sex^f^	Different to survivor	− 7.5	− 17.9	2.8	0.161	0.205	− 4.1	− 13.5	5.3	0.395	0.124
Sibling (corresponding) IQ (centred)	–	− 0.2	− 0.6	0.2	0.368	0.133	− 0.6	− 1.2	0.1	0.078	0.254
Sibling (corresponding) IQ^2^ (centred)	–	0.0	0.0	0.0	0.972	0.005	0.0	0.0	0.0	0.071	0.260
Survivor × sibling sex	Female:sib sex diff. inter	18.8	0.7	37.0	***0.047***	0.287	–	–	–	–	–
Survivor sex × radiotherapy	Female:therapy interaction	− 22.0	− 38.7	− 5.2	***0.013***	0.353	–	–	–	–	–

**Fig. 2:**
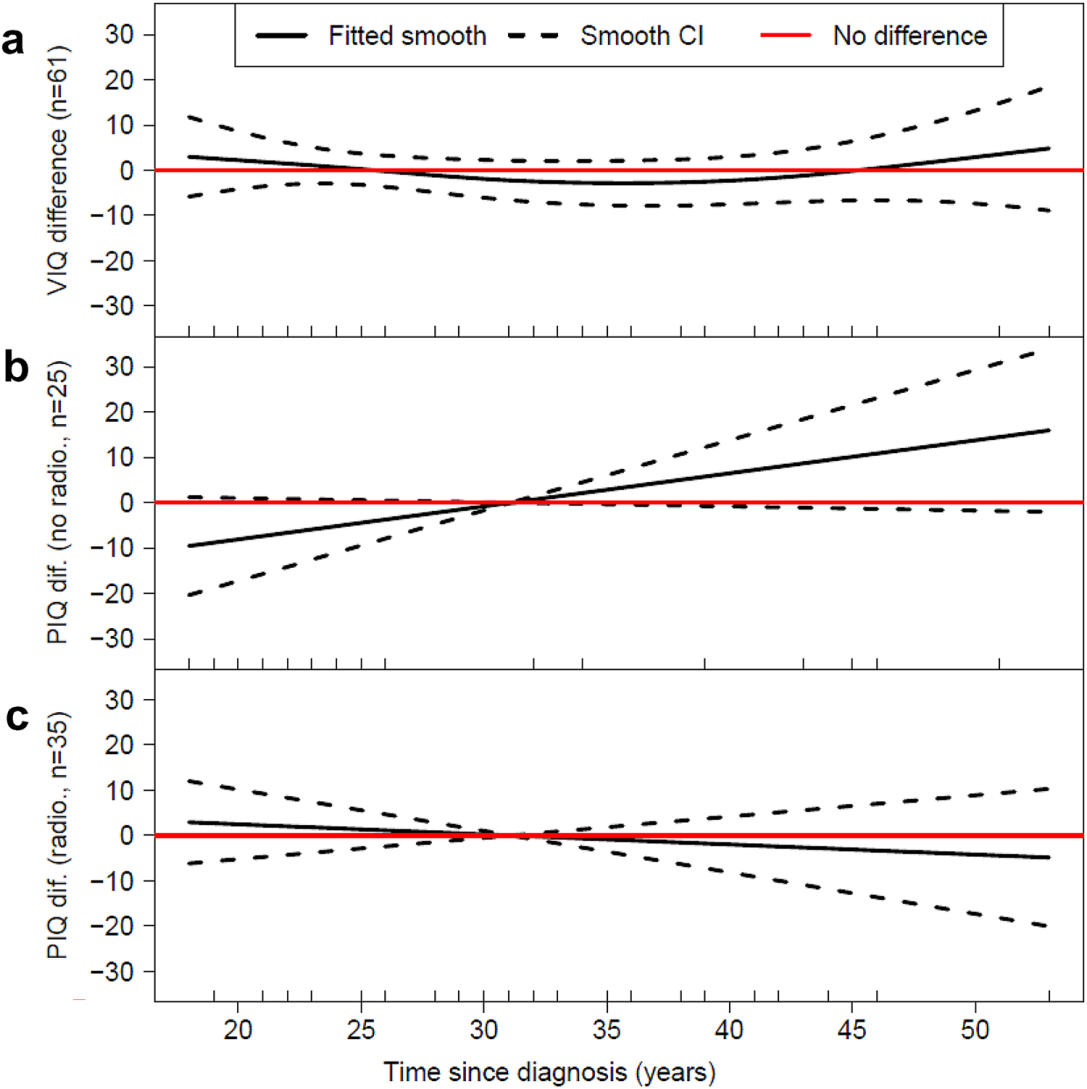
GAM smooth estimates for the relationship between time since diagnosis (years), while adjusting for all other covariates in the model in Table 3, and **a** verbal IQ (VIQ); **b** performance IQ (PIQ) among those with no confirmed radiotherapy; **c** PIQ among those with confirmed radiotherapy. The PIQ model includes an interaction between the smooth and radiotherapy, hence we have two smooths for PIQ **b** and **c**. The *y*-axis represents the difference between survivors’ and their siblings’ IQ scores; however, the difference shown must be combined with the effect of other covariates from Table 3 to be interpreted. Since survivor’s scores are, on average, lower than their siblings the difference in scores will be negative (survivor minus sibling will be less than zero); hence, a positive smooth value indicates the difference is decreased (ie survivors IQs are improving by getting closer to that of their siblings). Each panel includes a rug plot, showing the contributing participants/observations

The “Sibling (corresponding) IQ” is sibling VIQ in the VIQ model and sibling PIQ in the PIQ model. Cells only containing hyphens indicate terms not included in a model. Bold italics indicate a *p* value <0.05. Fits of the smooths of time since diagnosis included in the models are shown in Fig. 2

^a’^Medulloblastoma’ taken as reference level

^b’^No confirmed treatment’ taken as reference level

^c’^Absent’ taken as reference level

^d’^No recorded surgery’ taken as reference level

^e’^Men’ used as reference level

^f’^Same as survivor’ taken as reference level

A plot of the smooth showing the effect of time since diagnosis on VIQ difference is shown in Fig. 2 (top): there is no significant evidence (*p* = 0.5330) that this relationship differs from a horizontal line at zero (shown in the figure by the confidence region around the smooth being centred on the red line, plotted at zero difference). The fitted smooth has estimated degrees of freedom (EDF) of 1.8, approaching a quadratic relationship [matching the gentle ‘u’ shape seen in the Fig. 2 (top)]. This model does not include an interaction between the smooth (i.e. time since diagnosis) and radiotherapy: this suggests little evidence for an effect of radiotherapy on VIQ difference changing over time (otherwise a model including this interaction would have had a lower AICc and subsequently been selected).

Male survivors who had radiotherapy have VIQ scores that are on average 2.7 points closer to their siblings’ VIQ scores than male survivors who did not have radiotherapy (*b* = 2.7; 95% CI − 8.5, 13.8; *p* = 0.641). However, there is strong evidence (*p* = 0.013) that in female survivors, radiotherapy increases the VIQ difference between them and their siblings by around 19 points (− 22 + 2.7 ≈ 19; *b* = − 22.0; 95% CI − 38.7, − 5.2).

This analysis is repeated in the Supplementary Materials in a linear modelling framework, where the relationship between time since diagnosis and VIQ difference is restrictricted to a straight line. The model fits are very similar to those here and subsequent conclusions the same.

The Supplementary Materials also include a GAM as above, but extended to include two new covariates for additional years beyond compulsory education for survivors and siblings, respectively. Due to missing data on these covariates, the sample size reduces to *n* = 48. Conclusions are similar to the above primary analysis (time since diagnosis has no significant impact on VIQ; radiotherapy has a significant and larger impact on females).

### GAM: performance IQ (PIQ)

The PIQ GAM with lowest AICc is reported in Table 3 and includes one interaction (time since diagnosis × radiotherapy). In this model *n* = 60 (one participant has missing information about epilepsy and another is missing PIQ), adjusted *R*^2^ = 0.41 and diagnostic plots suggest no problems with model fit.

As there is an interaction between time since diagnosis and radiotherapy, a separate time since diagnosis relationship is estimated for those not receiving/receiving radiotherapy.

Figure 2 (middle) shows the estimated effect of time since diagnosis on PIQ on the n = 25 people who did *not* receive radiotherapy. A linear relationship (EDF = 1) is estimated, in which the PIQ difference increases (gets better) as time since diagnosis increases. This relationship approaches significance (*p* = 0.0822): the confidence interval region on Fig. 2 (middle) only just encompasses the line (red) of no difference.

Figure 2 (bottom) shows the estimated effect of time since diagnosis on PIQ on the *n* = 35 people who did receive radiotherapy. A linear relationship (EDF = 1) is estimated, in which the PIQ difference gets worse slightly as time since diagnosis increases. This relationship is not significant (*p* = 0.5235): the confidence interval region on Fig. 2 (bottom) is just off-centre of the line (red) of no difference.

Radiotherapy is associated, on average, with a significant (*p* < 0.001) and sizeable (*r* = 0.468) decrease (worsening) of 18 (*b* = − 18.0; 95% CI − 27.7, − 8.3) PIQ points (given the model parameterisation, this corresponds to the radiotherapy difference at approximately 31 years after diagnosis, the mean time since diagnosis). There is little evidence of this differing by sex, as the model does not include survivor sex × radiotherapy (otherwise a model including this interaction would have had a lower AICc).

This analysis is repeated in the Supplementary Materials in a linear modelling framework. As the estimated smooths are straight lines, the GAM and linear model are very similar, with the same conclusions.

The Supplementary Materials also include a GAM as above, but extended to include two new covariates for additional years beyond compulsory education for survivors and siblings respectively. Due to missing data on these covariates, the sample size reduces to *n* = 48. In contrast to the primary analysis above, there is no interaction between time since diagnosis and whether survivors receive radiotherapy. This difference is driven by two aspects of this secondary analysis which limit its wider applicability: first, the reduced sample size leads to a loss of statistical power to detect interactions; and second, there is a differential missingness on additional years beyond compulsory education and radiotherapy treatment—12% (3/26) among survivors not receiving radiotherapy, and 31% (11/36) among survivors who received radiotherapy. The differential missingness may induce a selection bias that we cannot quantify, and the loss of statistical power induces a model that averages across the two groups rather than producing group-specific estimates. As in the primary analysis above, radiotherapy significantly impacts on PIQ, but there is no evidence of a differential effect by sex.

## Discussion

This study investigated, in adult survivors of an early childhood PFT, effects on intellectual functioning of increasing time since tumour diagnosis. No previous studies of long-term neurocognitive outcomes of childhood PFT survivors have focused on the question investigated here: namely, is increasing time since diagnosis, continuing well into adulthood, associated with later changes in IQ. Intellectual functioning was assessed using the WASI index measures of VIQ and PIQ. In the absence of repeated IQ measures within individuals across time, in this study, VIQ and PIQ were measured on a single occasion in a cohort of survivors between 18 and 53 years after their tumour was diagnosed. Siblings’ IQ provided an estimated proxy for what individual survivors’ IQ might have been had they not developed tumours.

Results indicated that, compared to their siblings, older adult survivors of a childhood PFT did not have relatively lower VIQ and PIQ scores than younger adult survivors. However, relative IQ deficits did persist in adulthood, with the possible exception of PIQ scores in survivors who did not receive radiotherapy, in whom there was a trend towards a reduction in survivor–sibling PIQ difference with greater time since PFT diagnosis. Our results were broadly robust to the inclusion of covariates that measure educational levels (see Results—no improving trend for PIQ was found for survivors who did not receive radiotherapy, but this is likely driven by greater levels of missing data in the secondary analysis). Our data do not allow any conclusions to be drawn regarding the possible basis for differences between VIQ and PIQ over time. It may be relevant that in a recent study of children treated below the age of 4 years for a medulloblastoma, whose cognitive performance was assessed an average of 4.9 years after their surgery, it was noted that those who had received cranial–spinal irradiation were particularly impaired on motor decision time [26]. Further research will be needed to establish the time course of the effect of radiotherapy on this aspect of cognitive performance and to address the question of whether it relates to long-term differences between PIQ and VIQ outcomes.

Our findings indicating that following a childhood PFT relative cognitive deficits tend to persist is in line with other studies that have undertaken long-term follow-up in this clinical group. Schreiber et al. [27] noted that in male children followed for 5 years after surgery for a medulloblastoma, those whose acute treatment had been associated with the development of posterior fossa syndrome had relatively more impaired cognitive performance at one year post-surgery than those who did not develop it. Many of those impairments were still present 4 years later, and in some cases, declined further over the 4 years of follow-up. They concluded that early brain insult associated with posterior fossa syndrome may contribute to an acute decline in attention, processing speed, and working memory with very little recovery over time. They also concluded, given that most of their participants received low-dose radiation, that posterior fossa syndrome was a greater predictor of neurocognitive impairment than low-dose radiation. Posterior fossa syndrome is reported in up to 29% of those receiving surgery for medulloblastoma [28]. Whilst we do not have a figure for the prevalence of posterior fossa syndrome in the participants of the current study, it is likely to only have been present in a small proportion. Hence, whilst, like Schreiber et al. [27], we observed evidence suggesting the persistence across time of a range of cognitive deficits, in the population reported in the current study, it is unlikely that the explanation for this finding was the presence of a post-operative posterior fossa syndrome.

Considering findings from long-term follow-up studies, in one of the few previous studies that have followed survivors treated for a PFT in childhood into adulthood, Reimers et al. [13] reported IQ in participants with a mean age of 21.7 years and a mean age at diagnosis of 8.3 years. They observed a significant correlation between full-scale IQ and age at diagnosis but not between full-scale IQ and age at follow-up. These findings suggests, in results compatible with those from the current study, that IQ does not change progressively with increasing time since diagnosis. However, the inferences to be drawn from their study are limited by the wider age range at diagnosis of their participants and the much shorter period of follow-up into adulthood. Ellenberg et al. [11] examined adult cognitive outcomes in survivors of a childhood CNS malignancy. They observed a range of neurocognitive symptoms in adulthood, using a self-reported behavioural rating inventory, and did not examine potential effects of increasing intervals of time between original diagnosis and follow-up in adulthood.

Our results also showed that, regardless of time elapsed since tumour diagnosis, a history of receiving radiotherapy was associated with relatively greater lowering of VIQ scores in female survivors and greater lowering of PIQ scores in both male and female survivors. Including covariates for educational level led to the same conclusions. The association between a history of childhood radiotherapy and subsequent cognitive impairment has been much researched and reduced white matter integrity has been proposed as contributing to persisting deficits in neurocognitive function. This may particularly be the case given the role of white matter in cortical information processing and integration [29]. Links with damage to various white matter tracts have been reported [30, 31]. It has also been demonstrated recently that in addition to long-term effects of radiotherapy on cognition, functional impairments in cognition and diffuse white matter changes may be detected as soon as 3 months after treatment with wider-field, cranial–spinal radiotherapy compared to local radiotherapy [32]. Those authors also noted, however, that their group who had received just surgery, or surgery with local radiotherapy did over a subsequent follow-up period of 3 years also experience cognitive decline, although of lesser magnitude than those who had received cranial-spinal radiotherapy.

In a study that investigated white matter tract integrity in young adults a mean of 13 years after their brain tumour diagnosis, King et al. [31] reported an association between a history of radiotherapy, lower IQ at follow-up and disrupted white matter tract integrity. As noted by King et al. [31], the white matter disruption they detected could have reflected loss of white matter, delayed maturation of white matter, or individual vulnerability to neurotoxicity associated with the tumour or its management. The results of the present study, with data indicating a persisting detrimental effect of radiotherapy on IQ across participants who were assessed an average of 32.9 years after diagnosis, a considerably longer follow-up period than that reported by King et al. [31], suggests that of the possibilities proposed by King et al. [31], delayed maturation appears to be unlikely.

The reasons for the sex differences in the pattern of IQ results in those who received radiotherapy are unclear. Whilst no sex differences in intellectual outcomes have been reported in some studies [7], other reports do suggest that females may be more likely to have more adverse neurocognitive outcomes [33]. Ellenberg et al. [11], in a study of children and young people aged from 0–20 years when diagnosed with a central nervous system malignancy, reported that female sex predicted more impaired scores on two measures of self-reported neurocognitive outcome; Task Efficiency and Emotional Regulation scales, with small effect sizes. Ris et al. [15] on the other hand, in a study of children and young people aged 3–21 years with a medulloblastoma treated with radiotherapy and chemotherapy, found no effect of sex on intellectual outcome measured using WISC or WAIS IQ tests between 2 and 5 years after tumour diagnosis. Possible reasons for these differences in observed effects of sex on intellectual outcome may include treatments prevailing during the eras in which the participants were treated and the length of follow-up. The participants reported by Ris and colleagues were all treated after 1996, whilst those reported in the current study were treated between 1940 and 1991. Given the efforts to reduce radiation doses that have been pursued over time, it is possible that those reported here had received larger and less focused doses at a young age. However, the higher average age of tumour diagnosis and shorter follow-up period reported by Ris et al. [15] may also underpin the absence of observations of greater female vulnerability to adverse effects on IQ. Our finding of greater cognitive impairment in female survivors following a period of follow-up of at least 18 years, is congruent with the observation by Hudson et al. [34], in survivors of a wide range of cancers, that female survivors had a greater and steeper trajectory of decline in at least one health domain compared to their same sex sibling than did male survivors. It has also been noted that female survivors of acute lymphoblastic leukaemia are at increased risk for neurocognitive impairment, whether they received radiotherapy or chemotherapy [16]. There is also evidence that in non-cancer brain injury females may have worse outcomes that males [35, 36]. No specific mechanism underlying poorer cognitive outcomes in females after brain insult have been identified, though with respect to post-menarche females sustaining traumatic brain injury, it has been proposed that disrupted physiology of gonadal steroids, possibly through an effect on the anterior pituitary gland, may play a role [36].

In line with the North American Childhood Cancer survivor study, we chose to recruit a sibling comparison group, controlling for shared genetic and sociodemographic factors [37]. Previous research has demonstrated that the degree of hereditability for general intellectual functioning (*g*) is estimated as around 50% [38].

There are several limitations to our study. It is not longitudinal: instead of following survivors over time to examine changes in intellectual functioning, a cross-sectional design was used in which participants had a single IQ assessment. This design is limited by the age–period–cohort problem [39] and cannot separate effects of age-group, periods and cohorts; so cannot determine whether IQ changes relate to survivor age or to changes in treatment practices from 1940 to 1991. Additionally, our primary analysis does not include covariates that measure educational level—a quantity known to correlate with IQ—due to levels of missing data. However, we have conducted secondary analysis to check how our conclusions are impacted by variables that measure educational level: results are comparable given the reduced sample size.

Importantly, any study of treatment effects after follow-up periods of several decades will face the issue that treatment practices will have changed since the interventions were carried out. This does not invalidate the findings of the present study. First, this study provides information relevant to current adult survivors of an early childhood PFT, their families and clinicians, as to what may be expected with regard to cognitive performance during adulthood. Second, the current study provides a base for future follow-up studies of later cohorts of long-term survivors from clinical trials run during the 1990s and 2000s and who are now in their 20s and 30s, and comparison of our findings with data from such future studies would address the question of whether or not the observed pattern of long-term outcomes remain similar despite all the changes in treatment practices.

Our cognitive data are limited to estimates of VIQ and PIQ, meaning we cannot draw conclusions regarding specific aspects of cognition that would be of interest, for instance, processing speed. However, it is important to note that alternative opportunities for follow-up over as long as 50 years are limited.

Our observation that VIQ deficits, and PIQ deficits in those receiving radiotherapy, develop during childhood but persist, without further decline, in adulthood, suggests that the effects on intellectual functioning take the form of a fixed injury that imposes itself on subsequent cognitive development, rather than an ongoing pathological process. This emphasises the importance of seeking early interventions to optimize cognitive function during the developmental period [40].

## Electronic supplementary material

Below is the link to the electronic supplementary material.

Supplementary file1 (DOCX 51 kb)
