# Supplementary material for: Long-term cognitive outcome in adult survivors of an early childhood posterior fossa brain tumour
Source: Int J Clin Oncol. 2020 Jul 8;25(10):1763–73. doi: 10.1007/s10147-020-01725-7 (PMC7498491; doi:10.1007/s10147-020-01725-7)

**Supplementary materials**

**Comparison between survivors with and without sibling recruits**

Of the 118 participant survivors, 62 with a sibling who provided comparison data are reported on here. To provide an indication as to whether there were systematic differences between those survivors for whom a sibling provided data and the rest of the larger survivor group, we compared these two samples, finding small and non-significant differences in: presence of epilepsy (Fisher’s exact test, p=1.000; OR=0.980); time since diagnosis (t(114)=-0.860; p=0.392; r=0.080); age at diagnosis (t(113)=1.317; p=0.191; r=0.123); and tumour type (Fisher’s exact test, p=0.422). There was a medium size (OR=3.771, 95% CI: 0.924, 22.257) and just significant (Fisher’s exact test, p=0.047) difference in the proportion recorded as having had some form of surgery (in study – 82%, 51/62; not in study – 95%, 53/56). We do not consider further the survivors who did not have a participating sibling.

**Further information: Survivors: Diagnosis and treatment details – surgery**

There is no evidence of the age at diagnosis affecting the use of surgery (t(16)=0.004; p=0.997; r=0.001). However, those who received surgery had been diagnosed further into the past (mean time since diagnosis (years): surgery=32.0, no recorded surgery=27.9); this difference is statistically significant (t(46)=-2.189, p=0.033) and is a medium sized effect (r=0.308). There was little difference in the reported use of surgery across the different tumour types (surgery: medulloblastoma=87%, astrocytoma=82%, other=71%; Fisher’s exact test: p=0.772).

**Further information**: **IQ comparisons between survivors and siblings – subtests and sex comparisons**

Table S1 contains the WASI sub-test scores. One survivor could not complete the Block Design or Matrix Reasoning sub-test due to vision problems (and consequently has no PIQ score). On the T scores for each of the four sub-tests, survivors scored significantly lower than their siblings. The difference is around ten points on each test.

The unadjusted group IQ scores within each group are shown in Table S2.

**Further information: Linear Regression model: Verbal IQ**

The VIQ linear model with lowest AICc is reported in Table S4 and includes two interactions (survivor sex × sibling sex; survivor sex × radiotherapy). In this model n=61 (one participant has missing information about whether they have epilepsy), adjusted R²=0.30 and diagnostic plots suggest no problems with model fit.

There is no statistically significant (p=0.860) evidence of time since diagnosis affecting VIQ difference: each additional year increases VIQ difference (gets worse) by only 0.04 points per year (a very small effect: r=0.026). This model does not include an interaction between the smooth (fit to time since diagnosis) and radiotherapy: this suggests that there is little evidence of the effect of radiotherapy on VIQ difference changing over time (otherwise a model including this interaction would have had a lower AICc and have been selected).

Male survivors who had radiotherapy have VIQ scores that are on average 1.8 points closer to their siblings’ VIQ scores than males survivors who did not have radiotherapy (b=1.8; 95% CI: -9.7, 13.2; p=0.760). However, there is strong evidence (p=0.015) that in female survivors radiotherapy increases the VIQ difference between them and their siblings by around 20 points (-22+1.8≈20; b=-22.0; 95% CI=-39.5, -4.5). Since the interaction of time since diagnosis and radiotherapy is not significant, there is no evidence of the effect of radiotherapy on VIQ difference changing over time (time since diagnosis).

**Further information: Linear Regression model: Performance IQ (PIQ)**

The PIQ linear model with lowest AICc is reported in Table S4 and includes one interaction (time since diagnosis × radiotherapy). In this model n=60 (one participant has missing information about whether they have epilepsy and another is missing PIQ as noted above), adjusted R²=0.41 and diagnostic plots suggest no problems with model fit.

Given the time since diagnosis × radiotherapy interaction, we consider the effect of time since diagnosis on PIQ difference separately for those who did not receive and did receive radiotherapy.

Among those receiving *no* radiotherapy, the PIQ difference improves (gets better) on average by 0.7 PIQ points per year of time since diagnosis (b=0.7; 95% CI=-0.1, 1.6). This is a small-medium size effect (r=0.21) and approaches significance (p=0.082).

Among those *receiving* radiotherapy, the PIQ difference declines (gets worse) on average by 0.3 PIQ points per year of time since diagnosis (0.7+(-1.0) ≈-0.3; b=-1.0; 95% CI= -2.0, 0.1). The difference compared to those *not* receiving radiotherapy is a medium size effect (r=0.25) and approaches significance (p=0.084).

Radiotherapy is associated, on average, with a significant (p<0.001) and sizeable (r=0.470) decrease (worsening) of 18 (b=-18.2; 95% CI: -28.2, -7.3) PIQ points (given the model parameterisation, this corresponds to the radiotherapy difference at approximately 31 years after diagnosis, the mean time since diagnosis). There is little evidence of this differing by sex, as the model does not include survivor sex × radiotherapy (otherwise a model including this interaction would have had a lower AICc and have been selected).

**GAM: Verbal IQ (VIQ; including additional years beyond compulsory education covariate)**

The VIQ GAM with lowest AICc is reported in Table S5 and includes one interaction (survivor sex × radiotherapy). In this model n=48, adjusted R²=0.39 and diagnostic plots suggest no problems with model fit.

A plot of the smooth showing the effect of time since diagnosis on VIQ difference is shown in Figure S1 (top): there is no significant evidence (p=0.121) that this relationship differs from a horizontal line at zero (shown Figure S1 (top) by the confidence region around the smooth including the red line of zero difference). The fitted smooth has estimated degrees of freedom (EDF) of 1, giving a linear/straight line. This model does not include an interaction between the smooth (i.e. time since diagnosis) and radiotherapy: this suggests little evidence for an effect of radiotherapy on VIQ difference changing over time (otherwise a model including this interaction would have had a lower AICc and subsequently been selected).

Male survivors who had radiotherapy have VIQ scores that are on average 4.2 points closer to their siblings’ VIQ scores than male survivors who did not have radiotherapy (b=4.2; 95% CI: -5.6, 14.0; p=0.408). However, there is strong evidence (p=0.002) that in female survivors radiotherapy increases the VIQ difference between them and their siblings by around 23 points (-27.2+4.2≈-23; b=-27.2; 95% CI=-42.9, -11.4).

There were no significant associations between VIQ and either i) survivor additional years of education beyond compulsory education (p=0.080; b=1.6, 95% CI=-0.1, 3.4), nor ii) sibling additional years beyond compulsory education (p=0.147; b=1.4, 95% CI=-0.4, 3.3).

**GAM: Performance IQ (PIQ; including years of compulsory education covariate)**

The PIQ GAM with lowest AICc is reported in Table S5 and includes no interactions. In this model n=48, adjusted R²=0.45 and diagnostic plots suggest no problems with model fit.

A plot of the smooth showing the effect of time since diagnosis on VIQ difference is shown in Figure S1 (bottom): there is no significant evidence (p=0.286) that this relationship differs from a horizontal line at zero (shown in Figure S1 (bottom) by the confidence region around the smooth including the red line of zero difference). The fitted smooth has estimated degrees of freedom (EDF) of 1, giving a linear/straight line. This model does not include an interaction between the smooth (i.e. time since diagnosis) and radiotherapy: this suggests little evidence for an effect of radiotherapy on VIQ difference changing over time (otherwise a model including this interaction would have had a lower AICc and subsequently been selected).

Radiotherapy is associated, on average, with a significant (p<0.001) and sizeable (r=0.435) decrease (worsening) of 13 (b=-13.1; 95% CI: -22.3, -4.0) PIQ points (given the model parameterisation, this corresponds to the radiotherapy difference at approximately 31 years after diagnosis, the mean time since diagnosis). There is little evidence of this differing by sex, as the model does not include survivor sex × radiotherapy (otherwise a model including this interaction would have had a lower AICc).

There were no significant associations between PIQ and either i) survivor additional years of education beyond compulsory education (p=0.146; b=1.5, 95% CI:-0.5, 3.5), nor ii) sibling additional years beyond compulsory education (p=0.806; b=0.2, 95% CI:-1.7, 2.2).

**Table S1**:WASI sub-test scores in the survivors and siblings. Paired t-tests are used for comparing continuous variables in the 62 survivor and sibling pairs.

|  | **Survivors** | | | **Siblings** | | | **Survivor v. sibling (unadjusted) comparisons** |
| --- | --- | --- | --- | --- | --- | --- | --- |
| **Sub-Test** | **Mean** | **SD** | **Range** | **Mean** | **SD** | **Range** |  |
| Similarities | 42.6 | 12.4 | 20-61 | 52.1 | 7.5 | 35-68 | t(61)=-6.237, Dif=-9.5 (95% CI=-12.6, -6.5), p<0.001, r=0.624 |
| Vocabulary | 41.1 | 13.0 | 20-65 | 52.3 | 9.7 | 26-66 | t(61)=-6.328, Dif=-11.2 (95% CI=-14.8, -7.7), p<0.001, r=0.630 |
| Block Design | 45.5§ | 12.5 | 22-66 | 56.4 | 8.2 | 40-71 | t(60)=-6.301, Dif=-11.0 (95% CI=-14.5, -7.5), p<0.001, r=0.631 |
| Matrix Reasoning | 42.7§ | 15.3 | 20-70 | 55.6 | 7.7 | 31-72 | t(60)=-6.509, Dif=-13.0 (95% CI=-17.0, -9.0), p<0.001, r=0.643 |

§N=61.

**Table S2**: IQ scores by sex within each group. One survivor could not complete PIQ sub-tests due to vision problems, and so has no corresponding PIQ score.

| **Group** | **IQ type** | **Females** | | | **Males** | | | **Group comparisons** |
| --- | --- | --- | --- | --- | --- | --- | --- | --- |
|  |  | **n** | **Mean** | **SD** | **n** | **Mean** | **SD** |  |
| Survivors | VIQ | 24 | 81.9 | 19.4 | 38 | 92.4 | 16.2 | t(43)=-2.206, Dif=-10.5 (95% CI= -20.1, -0.9), p=0.033, r=0.320 |
|  | PIQ | 23 | 81.7 | 19.9 | 38 | 96.7 | 18.6 | t(44)=-2.922, Dif=-15.0 (95% CI=-25.3, -4.7), p=0.005, r=0.403 |
| Siblings | VIQ | 36 | 104.6 | 10.9 | 26 | 102.5 | 13.4 | t(47)=0.632, Dif=2.0 (95% CI= -4.4, 8.4), p=0.531; r=0.092 |
|  | PIQ | 36 | 109.1 | 12.2 | 26 | 111.1 | 12.6 | t(53)=-0.660, Dif=-2.1 (95% CI=-8.5, 4.3), p=0.512; r=0.090 |

**Table S3**: IQ scores by tumour, along with sibling comparisons using paired t-tests.

| **IQ type** |  | **Survivors** | | | **Siblings** | | | **Survivor v. sibling (unadjusted) comparisons** |
| --- | --- | --- | --- | --- | --- | --- | --- | --- |
|  | **Tumour type** | **Mean** | **SD** | **Range** | **Mean** | **SD** | **Range** |  |
| VIQ | Medulloblastoma (n=15) | 88.2 | 16.2 | 55-112 | 108.9 | 10.4 | 85-127 | t(14)=-3.944, Dif=-20.7 (95% CI=-31.9, -9.4), p=0.001, r=0.725 |
|  | Astrocytoma (n=40) | 88.0 | 18.7 | 55-119 | 101.5 | 12.4 | 77-122 | t(39)=-5.177, Dif=-13.5 (95% CI=-18.8, -8.2), p<0.001, r=0.637 |
|  | Other (n=7) | 90.4 | 21.1 | 55-121 | 105.1 | 10.5 | 90-122 | t(6)=-1.895, Dif=-14.7 (95% CI=-33.7, 4.3), p=0.107, r=0.612 |
| PIQ | Medulloblastoma (n=15) | 91.3 | 22.5 | 55-124 | 111.7 | 12.9 | 88-134 | t(14)=-3.165, Dif=-20.4 (95% CI=-34.2, -6.6), p=0.007, r=0.646 |
|  | Astrocytoma (n=40) | 90.6* | 19.3 | 56-129 | 108.8 | 12.4 | 83-134 | t(38)=-5.809, Dif=-18.5 (95% CI=-24.9, -12.0), p<0.001, r=0.686 |
|  | Other (n=7) | 93.1 | 24.0 | 60-125 | 113.3 | 11.0 | 96-128 | t(6)=-2.134, Dif=-20.1 (95% CI=-43.2, 3.0), p=0.077, r=0.657 |

*N=39.

**Table S4:** The fit of the *linear* regression models relating Verbal IQ (VIQ) and Performance IQ (PIQ) difference (survivor IQ-sibling IQ) to the covariates. The “Sibling (corresponding) IQ” is sibling VIQ in the VIQ model and sibling PIQ in the PIQ model. Grey cells indicate terms not included in a model. ***Bold italics*** indicate a p-value<0.05.

|  |  | **VIQ (n=61; adjusted R²=0.30)** | | | | | **PIQ (n=60; adjusted R²=0.41)** | | | | |
| --- | --- | --- | --- | --- | --- | --- | --- | --- | --- | --- | --- |
| **Variable** | **Categorical level** | **b** | **95% CI** | | **P-value** | **r** | **b** | **95% CI** | | **P-value** | **r** |
| Intercept | - | -19.1 | -41.4 | 3.1 | 0.09 | 0.245 | 0.1 | -24.3 | 24.5 | 0.993 | 0.001 |
| Time since diagnosis (years) | - | 0.0 | -0.5 | 0.4 | 0.86 | 0.026 | 0.7 | -0.1 | 1.6 | 0.082 | 0.251 |
| Age at diagnosis (months) | - | -0.2 | -0.5 | 0.2 | 0.355 | 0.135 | 0.3 | 0.0 | 0.7 | 0.074 | 0.258 |
| Tumour type* | Astrocytoma | 4.6 | -6.4 | 15.6 | 0.408 | 0.121 | -3.0 | -15.2 | 9.2 | 0.62 | 0.073 |
|  | Other | 11.0 | -4.2 | 26.2 | 0.153 | 0.207 | -5.0 | -22.6 | 12.6 | 0.572 | 0.083 |
| Radiotherapy† | Confirmed radiotherapy | 1.8 | -9.7 | 13.2 | 0.761 | 0.045 | -18.2 | -28.2 | -8.2 | <0.001 | 0.470 |
| Epilepsy‡ | Present | -7.5 | -18.2 | 3.1 | 0.161 | 0.203 | -9.5 | -21.6 | 2.5 | 0.117 | 0.227 |
| Surgery§ | Confirmed surgery | 16.5 | 4.6 | 28.3 | ***0.007*** | 0.378 | 4.3 | -8.1 | 16.6 | 0.491 | 0.101 |
| Survivor sexǁ | Female | -3.1 | -17.2 | 11.0 | 0.659 | 0.065 | -16.8 | -26.2 | -7.3 | ***<0.001*** | 0.461 |
| Sibling sex¶ | Different to survivor | -7.2 | -18.1 | 3.6 | 0.186 | 0.192 | -4.1 | -13.7 | 5.5 | 0.395 | 0.124 |
| Sibling (corresponding) IQ (centred) | - | -0.2 | -0.6 | 0.2 | 0.344 | 0.138 | -0.6 | -1.2 | 0.1 | 0.078 | 0.254 |
| Sibling (corresponding) IQ² (centred) | - | 0.0 | 0.0 | 0.0 | 0.915 | 0.016 | 0.0 | 0.0 | 0.0 | 0.071 | 0.260 |
| Survivor× sibling sex | Female:sib sex diff. inter. | 18.6 | -0.3 | 37.5 | ***0.054*** | 0.277 | - | - | - | - | - |
| Survivor sex × radiotherapy | Female:therapy interaction | -22.0 | -39.5 | -4.5 | ***0.015*** | 0.346 | - | - | - | - | - |
| Time since diagnosis × radiotherapy | Time:therapy interaction | - | - | - | - | - | -1.0 | -2.0 | 0.1 | 0.084 | 0.250 |

|  |  |  |
| --- | --- | --- |
| *'Medulloblastoma' taken as reference level. | †`No confirmed treatment' taken as reference level. | ‡'Absent' taken as reference level. |
| §`No recorded surgery' taken as reference level. | ǁ'Male' used as reference level. | ¶'Same as survivor' taken as reference level. |

**Table S5:** The fit of the generalised additive models (GAMs) relating Verbal IQ (VIQ) and Performance IQ (PIQ) difference (survivor IQ-sibling IQ) to the covariates among survivor/sibling pairs with complete data on years beyond compulsory education. The “Sibling (corresponding) IQ” is sibling VIQ in the VIQ model and sibling PIQ in the PIQ model. Grey cells indicate terms not included in a model. ***Bold italics*** indicate a p-value<0.05. Fits of the smooths of time since diagnosis included in the models are shown in Figure S1.

|  |  | **VIQ (n=48; adjusted R²=0.39)** | | | | | | | **PIQ (n=48; adjusted R²=0.45)** | | | | | | | | | |
| --- | --- | --- | --- | --- | --- | --- | --- | --- | --- | --- | --- | --- | --- | --- | --- | --- | --- | --- |
| **Variable** | **Categorical level** | **b** | **95% CI** | | **P-value** | | **r** | | **b** | | **95% CI** | | | | **P-value** | | **r** | |
| Intercept | - | -29.5 | -51.4 | -7.7 | 0.012 | | 0.419 | | -13.7 | | -38.0 | | 10.6 | | 0.277 | | 0.186 | |
| Age at diagnosis (months) | - | -0.1 | -0.4 | 0.2 | 0.466 | | 0.127 | | 0.2 | | -0.1 | | 0.6 | | 0.173 | | 0.232 | |
| Tumour type* | Astrocytoma | 6.4 | -5.1 | 17.9 | 0.282 | | 0.187 | | 1.2 | | -11.4 | | 13.8 | | 0.852 | | 0.032 | |
|  | Other | 16.0 | 0.5 | 31.4 | 0.051 | | 0.333 | | 11.7 | | -5.5 | | 28.9 | | 0.190 | | 0.223 | |
| Radiotherapy† | Confirmed radiotherapy | 4.2 | -5.6 | 14.0 | 0.408 | | 0.144 | | -13.1 | | -22.3 | | -4.0 | | ***<0.001*** | | 0.435 | |
| Epilepsy‡ | Present | 8.2 | -4.8 | 21.3 | 0.225 | | 0.210 | | 1.2 | | -12.7 | | 15.0 | | 0.870 | | 0.028 | |
| Surgery§ | Confirmed surgery | 9.7 | -0.7 | 20.2 | 0.077 | | 0.303 | | 2.5 | | -8.8 | | 13.8 | | 0.669 | | 0.074 | |
| Survivor sexǁ | Female | 4.8 | -6.4 | 15.9 | 0.408 | | 0.144 | | -19.9 | | -28.2 | | -11.6 | | ***<0.001*** | | 0.629 | |
| Sibling sex¶ | Different to survivor | 0.9 | -6.8 | 8.6 | 0.822 | | 0.039 | | 3.0 | | -5.6 | | 11.6 | | 0.504 | | 0.115 | |
| Sibling (corresponding) IQ (centred) | - | -0.7 | -1.1 | -0.2 | ***0.008*** | | 0.438 | | -0.6 | | -1.3 | | 0.1 | | 0.096 | | 0.282 | |
| Sibling (corresponding) IQ² (centred) | - | 0.0 | 0.0 | 0.0 | 0.744 | | 0.057 | | 0.0 | | 0.0 | | 0.0 | | 0.766 | | 0.051 | |
| Survivor additional years beyond compulsory education | - | 1.6 | -0.1 | 3.4 | 0.080 | | 0.300 | | 1.5 | | -0.5 | | 3.5 | | 0.146 | | 0.247 | |
| Sibling additional years beyond compulsory education | - | 1.4 | -0.4 | 3.3 | 0.147 | | 0.251 | | 0.2 | | -1.7 | | 2.2 | | 0.806 | | 0.042 | |
| Survivor sex × radiotherapy | Female:therapy interaction | -27.2 | -42.9 | -11.4 | | ***0.002*** | | 0.508 | |  | |  | |  | |  | |  |

|  |  |  |
| --- | --- | --- |
| *'Medulloblastoma' taken as reference level. | †`No confirmed treatment' taken as reference level. | ‡'Absent' taken as reference level. |
| §`No recorded surgery' taken as reference level. | ǁ'Men' used as reference level. | ¶'Same as survivor' taken as reference level. |

**Figure S1:** GAM smooth estimates for the relationship between time since diagnosis (years), while adjusting for all other covariates in the model in Table S5, and: a) verbal IQ (VIQ); b) performance IQ (PIQ). The y-axis represents the difference between survivors’ and their siblings’ IQ scores, however the difference shown must be combined with the effect of other covariates from Table S5 to be interpreted. Since survivor’s scores are, on average, lower than their siblings the difference in scores will be negative (survivor minus sibling will be less than zero); hence a positive smooth value indicates the difference is decreased (ie survivors IQs are improving by getting closer to that of their siblings). Each panel includes a rug plot, showing the contributing participants/observations.


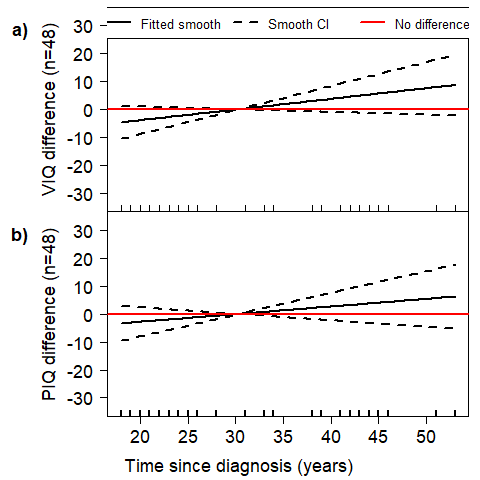

Supplement: Supplementary file 1 — Supplementary file1 (DOCX 51 kb) [file 10147_2020_1725_MOESM1_ESM.docx]
